# Supplementary material for: Hydrogen/Deuterium Exchange Mass Spectrometry for Probing the Isomeric Forms of Oleocanthal and Oleacin in Extra Virgin Olive Oils
Source: Molecules. 2023 Feb 22;28(5):2066. doi: 10.3390/molecules28052066 (PMC10004237; doi:10.3390/molecules28052066)
Supplement: Supplementary file 1 [file molecules-28-02066-s001.zip › molecules-2223083-supplementary.pdf]

Supplementary Materials

Hydrogen/Deuterium exchange mass spectrometry for probing the isomeric forms of oleocanthal and oleacin in extra virgin olive oils

R. Abbattista[a,&] , I. Losito*[,a,b], G. Basile[a], A. Castellaneta[a], G. Ventura[a,b], C.D. Calvano[a,b], T.R.I. Cataldi[a,b]

[a]Dipartimento di Chimica and [b]Centro Interdipartimentale SMART, Università degli Studi di Bari Aldo Moro, via Orabona 4, 70126 Bari, Italy

[&]Current address: Department of Plant Sciences, University of California, Davis, One Shields Avenue, 95616 Davis, CA, USA

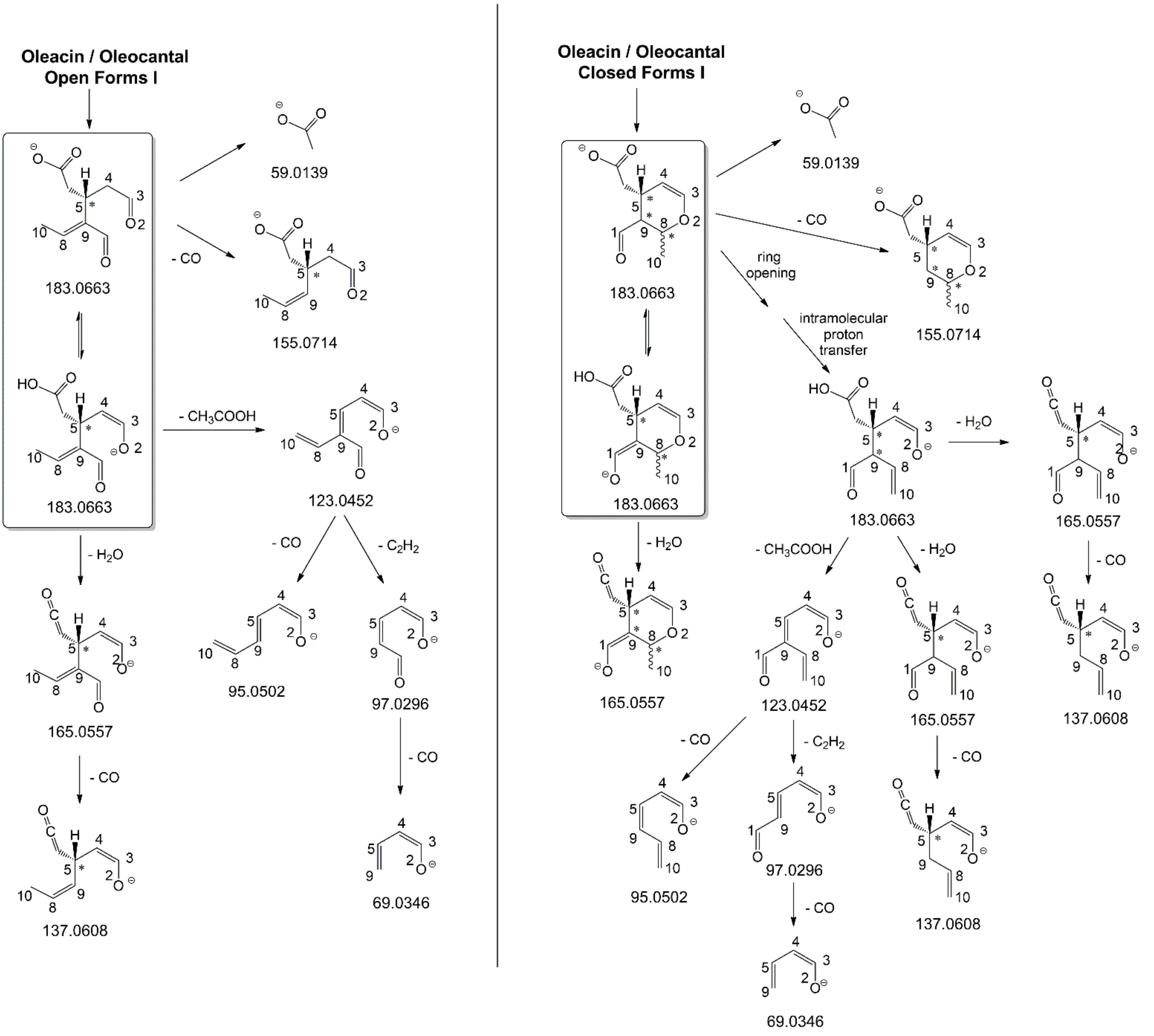

Figure S1. Fragmentation pathways hypothesised to explain the generation of product ions detected in MS/MS spectra referred to oleacin and oleocanthal chromatographic peaks (see Figures 2B and 3B in the main manuscript), starting from their common leading product ion, *i.e.*, the anion of decarboxymethyl-elenolic acid (exact m/z 183.0663). Pathways proposed for *Open Forms I* and *Closed Forms I* hypothesised for the two secoiridoids (see Figure 1 in the main manuscript) are shown.

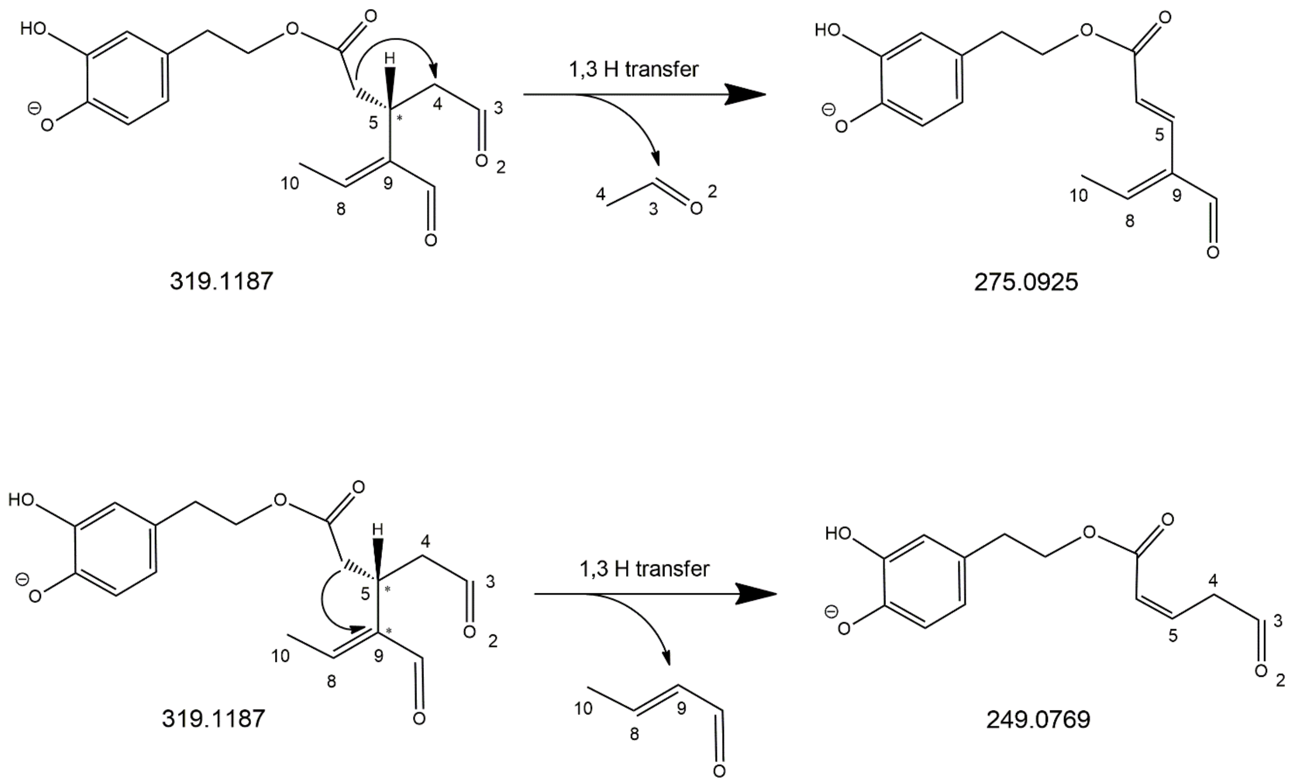

Figure S2. Fragmentations hypothesised to explain the detection of product ions consistent with the exact *m/z* ratios 275.0925 and 249.0769 upon fragmentation of the anions of oleacin isoforms, whose exact *m/z* ratio is also reported. Note that the process is shown only for *Open Forms I* of oleacin, although it may occur also for *Open Forms II* and *Closed Forms I* (see the main manuscript for details).

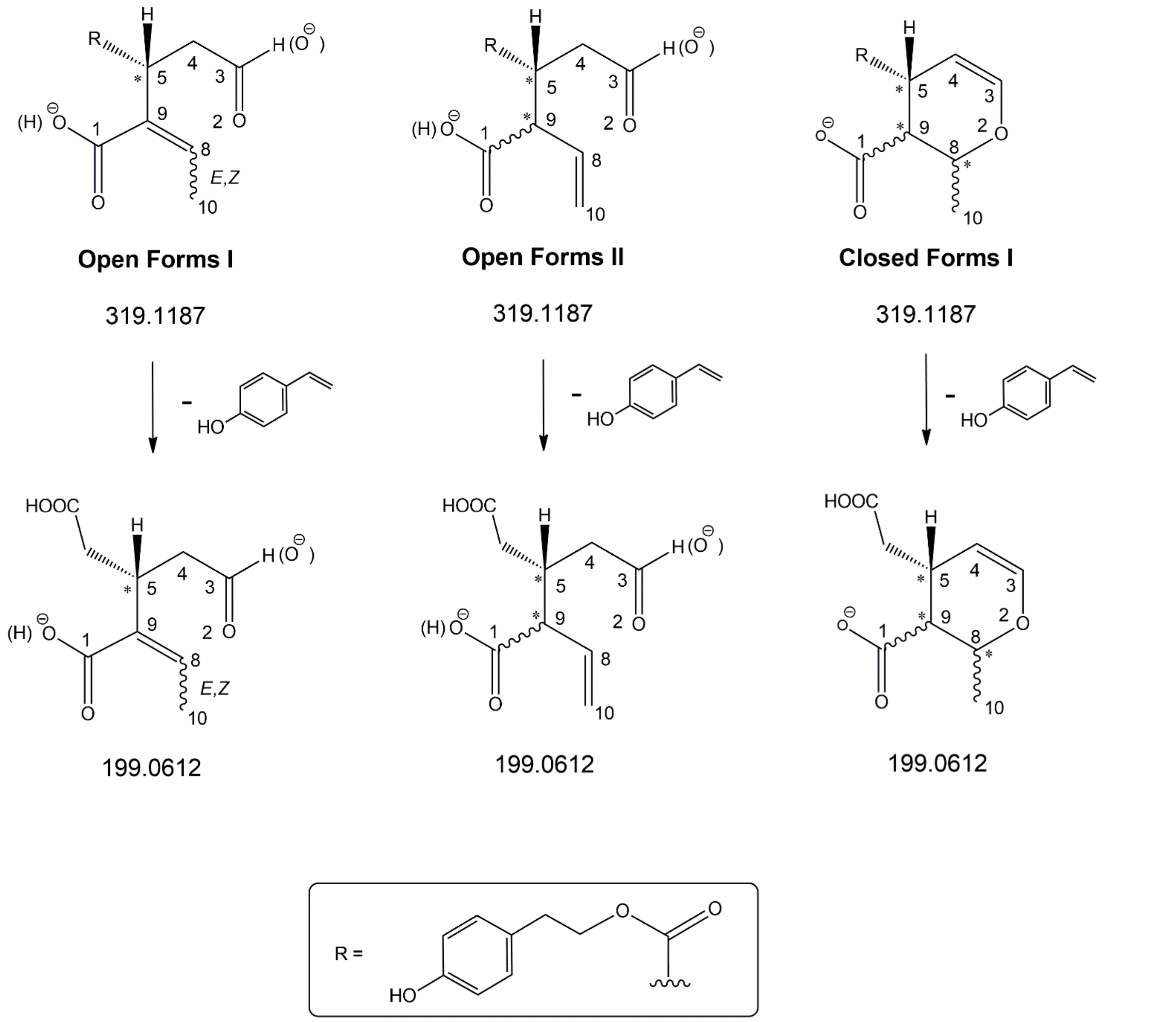

Figure S3. Representation of the fragmentation pathway (neutral loss of dehydrated tyrosol) leading from the anions of different isoforms of oleocanthalic acid (detected in the same XIC trace of oleacin, being isomeric with it) to the main product ion observed in the corresponding MS/MS spectra.

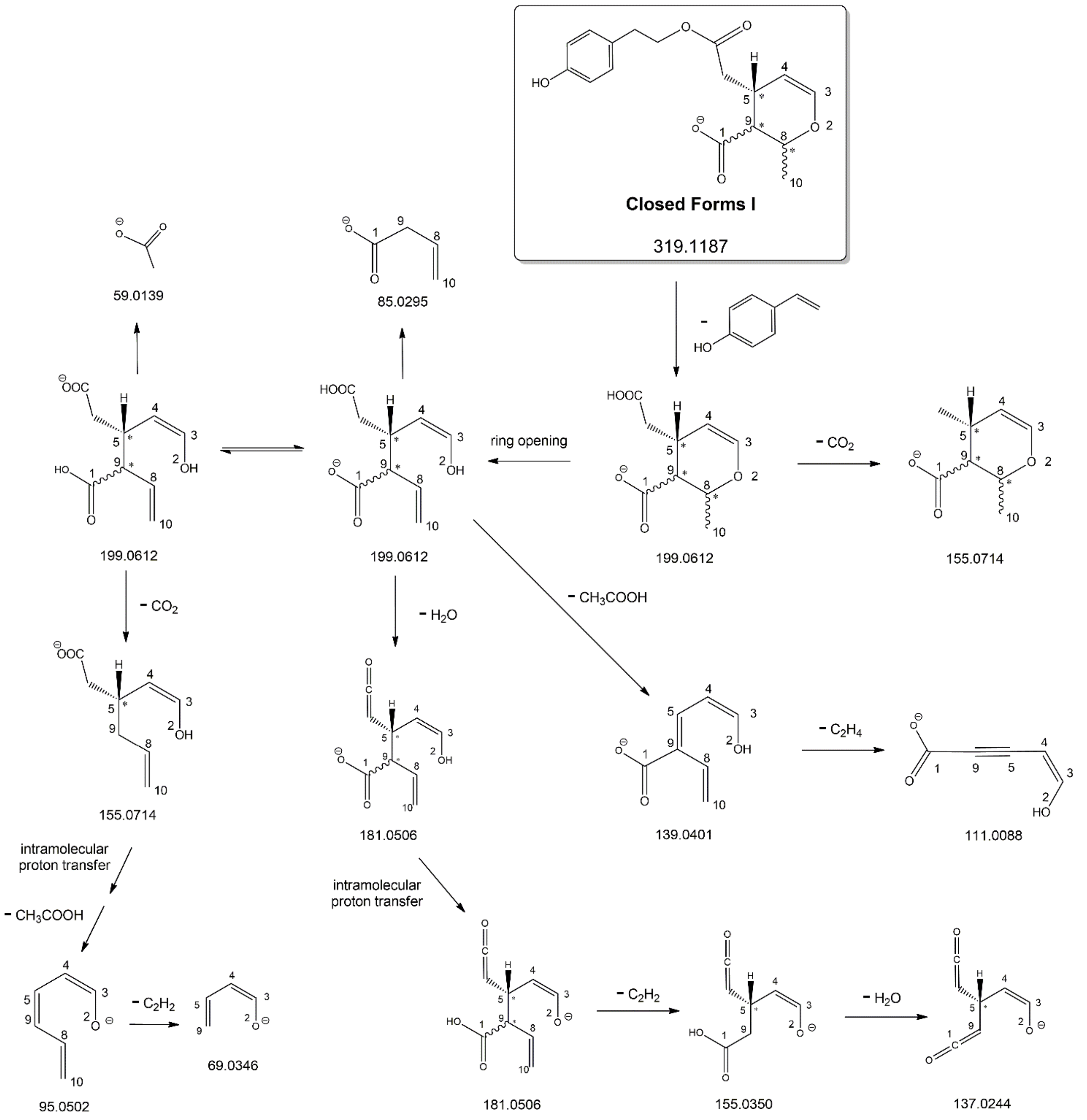

Figure S4. Fragmentation pathways hypothesised to explain product ions detected in MS/MS spectra of oleocanthalic acid *Closed Forms I*, starting from the main product ion observed in the corresponding MS/MS spectra (*m/z* 199.0612).

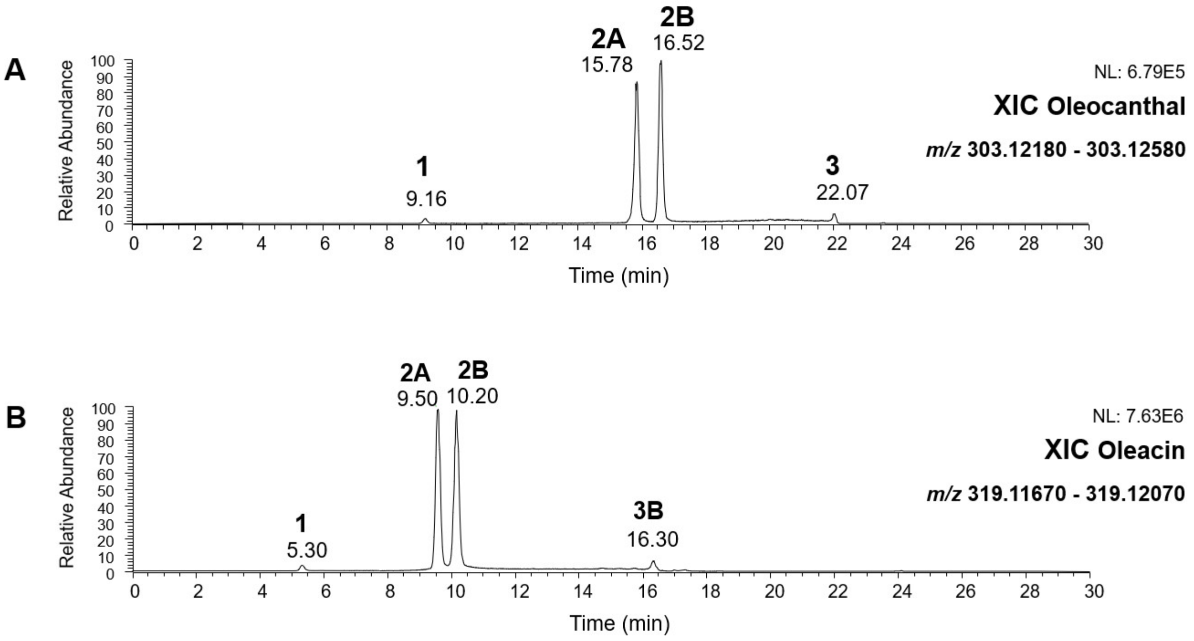

Figure S5. Extracted Ion Current (XIC) chromatograms referred to monoisotopic deprotonated oleocanthal (A, *m/z* 303.1238) and oleacin (B, *m/z* 319.1187) obtained after the RPLC-ESI(-)-FTMS analysis of a solution of commercial standards (phyproof® Reference Substances) of the two secoiridoids, each at a 10 mg/L concentration.

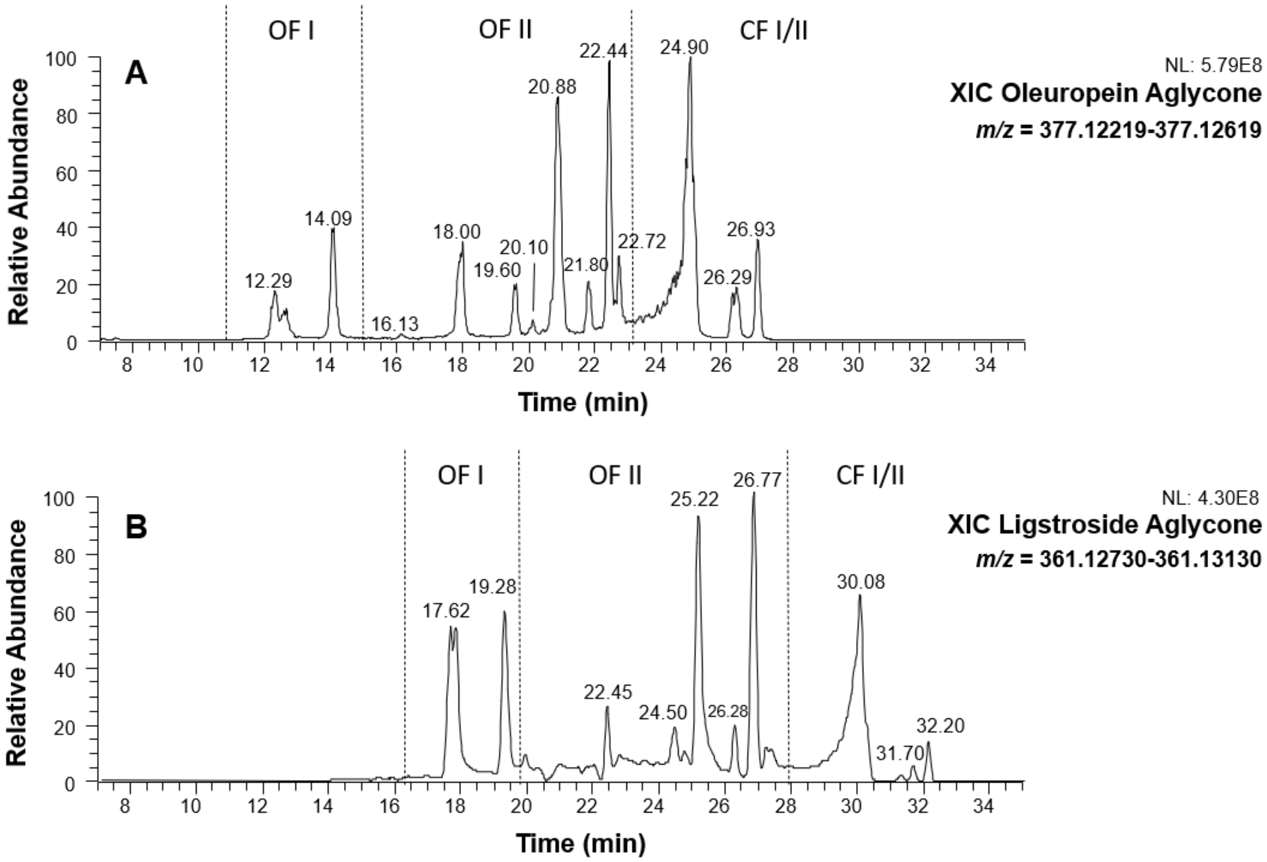

Figure S6. Extracted Ion Current (XIC) chromatograms referred to monoisotopic deprotonated oleuropein aglycone (A, *m/z* 377.1224) and ligstroside aglycone (B, *m/z* 361.1293) obtained after the RPLC-ESI(-)-FTMS analysis of the extract obtained from the EVOO considered during the present study. Peaks referred to isoforms of the same type (*Open Forms I/II*, OF I/OF II, and *Closed Forms I/II*, CF I/CFII; see Figure 1 in the manuscript) are separated by dotted lines.
